# Epistatic role of base excision repair and mismatch repair pathways in mediating cisplatin cytotoxicity

**DOI:** 10.1093/nar/gkt479

**Published:** 2013-06-12

**Authors:** Anbarasi Kothandapani, Akshada Sawant, Venkata Srinivas Mohan Nimai Dangeti, Robert W. Sobol, Steve M. Patrick

**Affiliations:** ^1^Department of Biochemistry and Cancer Biology, University of Toledo – Health Science Campus, Toledo, OH 43614, USA, ^2^Department of Pharmacology & Chemical Biology, University of Pittsburgh School of Medicine, Pittsburgh, PA 15213, USA, ^3^University of Pittsburgh Cancer Institute, Hillman Cancer Center, Pittsburgh, PA 15213, USA and ^4^Department of Human Genetics, University of Pittsburgh Graduate School of Public Health, Pittsburgh, PA 15213, USA

## Abstract

Base excision repair (BER) and mismatch repair (MMR) pathways play an important role in modulating cis-Diamminedichloroplatinum (II) (cisplatin) cytotoxicity. In this article, we identified a novel mechanistic role of both BER and MMR pathways in mediating cellular responses to cisplatin treatment. Cells defective in BER or MMR display a cisplatin-resistant phenotype. Targeting both BER and MMR pathways resulted in no additional resistance to cisplatin, suggesting that BER and MMR play epistatic roles in mediating cisplatin cytotoxicity. Using a DNA Polymerase β (Polβ) variant deficient in polymerase activity (D256A), we demonstrate that MMR acts downstream of BER and is dependent on the polymerase activity of Polβ in mediating cisplatin cytotoxicity. MSH2 preferentially binds a cisplatin interstrand cross-link (ICL) DNA substrate containing a mismatch compared with a cisplatin ICL substrate without a mismatch, suggesting a novel mutagenic role of Polβ in activating MMR in response to cisplatin. Collectively, these results provide the first mechanistic model for BER and MMR functioning within the same pathway to mediate cisplatin sensitivity via non-productive ICL processing. In this model, MMR participation in non-productive cisplatin ICL processing is downstream of BER processing and dependent on Polβ misincorporation at cisplatin ICL sites, which results in persistent cisplatin ICLs and sensitivity to cisplatin.

## INTRODUCTION

cis-Diamminedichloroplatinum (II) (cisplatin) has been widely used as a chemotherapeutic agent to treat a variety of cancers such as testicular, ovarian, lung and head and neck cancers. Cisplatin, like many chemotherapeutic drugs, targets DNA. The resulting inhibition of DNA replication and transcription results in the induction of apoptosis, which ultimately results in cancer regression (1). Cisplatin forms a variety of DNA adducts including monoadducts, intrastrand adducts and interstrand cross-links (ICLs) between two complementary DNA strands (2). The different types of DNA lesions bend and distort the DNA structure in a unique manner and are recognized and repaired differently. The monoadducts and intrastrand DNA lesions are repaired via the nucleotide excision repair (NER) pathway (3). Cells use multiple mechanisms to repair ICLs including NER, homologous recombination, the Fanconi Anemia pathway and lesion bypass mechanisms (4–7). These pathways can be both error free and error prone in nature. The exact mechanisms and biochemical events that occur during ICL DNA repair are still not completely understood.

A possible role of the DNA mismatch repair (MMR) pathway in cisplatin DNA adduct repair or damage tolerance has been suggested (8,9). MMR functions in the post-replicative repair of single-base mismatches and small insertion/deletion loops generated during DNA replication and thus helps maintain genomic integrity (10). The mismatches are recognized by one of the two complexes: hMutSα (heterodimer of human MutS homologues, hMSH2 and hMSH6) or hMutSβ (heterodimer of hMSH2 and hMSH3), which recruit hMutLα (heterodimer of hMLH1 and hPMS2) or hMutLβ (heterodimer of hMLH1 and hPMS1) complexes. MMR proteins are also necessary for activating cell cycle check points and pro-apoptotic signaling in response to certain DNA-damaging agents (11). DNA adducts formed by alkylating agents and 6-thioguanine form substrates for the MMR machinery and the MMR system repairs the mispaired alkylated bases (12). However, MMR deficiency causes resistance to the cytotoxic effects of certain DNA-damaging agents including cisplatin (13). It is well established that loss of MMR results in low-level resistance to cisplatin (14–19). Several hypotheses have been postulated on the mechanism of MMR-mediated cisplatin resistance including a ‘futile cycle’ of repair of cisplatin intrastrand damaged DNA, apoptotic signaling and a repair shielding model (8,20–24). Inactivation of MMR would be detrimental for any of these proposed models and lead to cisplatin resistance (25). A common feature in these models is the binding of the MMR proteins to the intrastrand adducts. However, the interaction of MMR proteins with cisplatin ICLs is poorly understood. *Escherichia coli* MutS binds to cisplatin ICLs, but with much less affinity compared with intrastrand adducts (26). Zhu and Lippard (27) demonstrated that hMutSβ binds to cisplatin ICLs by photo-cross-linking methods and electrophoretic mobility shift assays (EMSA). The macromolecular events that occur at cisplatin ICLs resulting in the binding of MMR damage recognition proteins remain unexplored.

Studies by us and others indicate that the base excision repair (BER) pathway modulates cisplatin sensitivity (28–30). Polymerase β (Polβ) is capable of bypassing and synthesizing past cisplatin intrastrand adducts, which might lead to cisplatin DNA-damage tolerance and increased mutagenicity (7,31). Using synthetic DNA oligonucleotides, we have recently shown that cisplatin ICLs can also be substrates for the BER machinery and that Polβ has low fidelity at the DNA flanking a cisplatin ICL (30). Polβ-mediated misincorporation of nucleotides at sites flanking the cisplatin ICL would result in mismatched bases, which could act as a nucleation point for the MMR pathway. Therefore, we targeted BER and MMR pathways separately and together to assess the roles these pathways play in mediating cisplatin sensitivity. Using isogenic mouse embryonic fibroblasts (MEFs) and human cancer cells, we show that BER and MMR pathways have overlapping roles in mediating cisplatin sensitivity. We also show that MMR acts downstream of BER, and that activation of MMR is dependent on Polβ misincorporation at cisplatin ICL sites. Based on these results, we propose a model in which BER and MMR processing of cisplatin ICL DNA leads to non-productive repair of the cisplatin ICLs and the resulting persistent ICLs mediate cisplatin sensitivity. This is the first evidence that BER and MMR pathways play an epistatic role in mediating cisplatin sensitivity.

## MATERIALS AND METHODS

### Chemicals and reagents

Cisplatin and methoxyamine (MX) were purchased from Sigma-Aldrich. All other chemicals and reagents were from standard suppliers. Antibody directed against MSH2 was from Calbiochem (NA27) and α-tubulin was from Sigma-Aldrich.

### Cell lines

The human breast adenocarcinoma MDA-MB-231 cells were grown in RPMI 1640 containing 10% heat-inactivated FBS and gentamycin (10 μg/ml). MDA-MB-231 Polβ knockdown cells (Polβ lentiviral shRNA) were grown in the presence of 0.5 μg/ml puromycin. MDA-MB-231 Polβ knockdown (KD) cells re-expressing wild-type Polβ and a variant deficient in polymerase activity (D256A) were grown under similar conditions with the addition of 700 μg/ml geneticin. The development and characterization of the different MDAMB-231 cells were described previously (32). Wild-type (92TAg) and Polβ null (88TAg) primary MEFs were cultured in high glucose Dulbecco’s modified Eagle’s medium supplemented with 10% fetal bovine serum (FBS) plus antibiotics at 37°C in a humidified atmosphere under 10% CO_2_. The hMSH2-deficient human endometrial adenocarcinoma cell line (Hec59) and subline complemented with chromosome 2 (Hec59+2) were grown in Dulbecco’s modified Eagle’s medium/F12. The chromosome complemented cell lines were maintained in medium supplemented with geneticin (400 µg/ml). The MMR proficient and deficient cells were kindly provided by Dr. Kandace Williams, University of Toledo Medical Center.

### siRNA transfection

ON-TARGETplus SMART pool siRNAs designed to target human and mouse MSH2 and control siRNA (ON-Targetplus Non-Targeting Pool) were purchased from Dharmacon RNAi Technologies, Thermo Scientific. Cells were seeded in 6-well plates, and two transfections were done at 24 h intervals. Tranfections were carried out as per the manufacturer’s instruction using specific DharmaFECT transfection reagents for each cell line.

MEFs (wild-type and Polβ null) and MDA-MB-231 (wild-type and Polβ knockdown) cells were transfected with siRNA directed against MSH2. The cells were harvested at indicated time points and analyzed for protein expression (Supplementary Figure S1). We observed a significant downregulation of MSH2 at 48 and 72 h in MEFs (A,B) as well as MDA-MB-231 cells (C,D). Quantification of transcript levels showed that we achieved >90% knockdown in MEFs and ∼75% knockdown in MDA-MB-231 cells (Supplementary Figure S2).

### Colony survival assay

Cells were (∼400) treated with increasing concentrations of cisplatin for 2 h. MX was added 2 h before cisplatin treatment. After treatment, fresh medium was added, and the cells were allowed to grow for 7–14 days. Colonies were fixed with 95% methanol and stained with 0.2% crystal violet. Colonies with ≥50 cells were counted, and colony survival was expressed as the ratio of the average number of colonies in drug-treated cells versus control cells × 100. The experiment was done in triplicates for each drug concentration.

### Cisplatin interstrand cross-link measurement

Modified alkaline comet assay was used to analyze the repair of cisplatin ICLs as described (33,34). Cell suspensions (∼10 000 cells) were embedded on a microscopic slide, lysed and incubated in ice-cold alkaline solution for 20 min. Electrophoresis was carried out for 25 min at 28 V, 300 mA. Slides were neutralized and stained with SYBR green (Trevigen). The comets were scored using a Nikon epifluorescence microscope. At least 50 cells were analyzed per slide using Komet Assay Software 5.5F (Kinetic Imaging, Liverpool, UK). The data were expressed as the percentage of cross-links that remained at that particular time point normalized to 100% at 0 h.

### Biotin–DNA pull downs

Biotinylated duplex DNAs either undamaged or containing a single mismatch, a single cisplatin ICL or a cisplatin ICL with a mismatch were synthesized as described previously (30,35). The substrates were bound to streptavidin magnetic beads (Dynabeads, Dynal Biotech) in the presence of binding buffer [20 mM HEPES (pH 7.8), 2 mM DTT, 0.001% NP-40, 100 mM NaCl and 200 mM MgCl_2_] at 4°C for 30 min. The beads were washed three times with binding buffer to remove all the unbound DNA substrates. Next, 5 µg of hMSH2-hMSH6 baculovirus infected SF-9 insect cell extract was added with 30-fold excess of poly dI-dC competitor DNA. The tubes were rotated for 1 h at 4°C. All the tubes were kept in a magnetic separation stand (Promega). The tubes were then washed three times in wash buffer (binding buffer with 200 mM NaCl), followed by elution using 1M NaCl. The tubes were left to rotate for 30 min and again placed in a magnetic separation stand, and the supernatants were collected and trichloroacetic acid (TCA) precipitated. The resulting pellets were resuspended in binding buffer and loaded onto 8% SDS gels, transferred to PVDF membrane and probed with MSH2 (Calbiochem) antibody. A similar experimental protocol was followed for the undamaged and cisplatin ICL substrate containing a uracil adjacent to the cross-link except that it was treated with uracil DNA glycosylase (UDG), Apurinic endonuclease (Ape1) and DNA Polβ in the presence of all the dNTPs. This BER processed DNA generates a substrate with either a correct or incorrect base adjacent to the cisplatin ICL (30). The undamaged and cisplatin ICL DNA substrates were recovered by ethanol precipitation and used for the Biotin–DNA–streptavidin pull down experiments in Figure 5C.

### EMSA analysis

Radiolabeled 42-mer DNA substrates (undamaged, undamaged containing a mismatch, a single cisplatin ICL, a single cisplatin ICL with a mismatch) were synthesized as described previously (30). These substrates were labeled at the 5′ terminus with [γ-^32^P] ATP using T4 polynucleotide kinase. The mobility shifts were conducted according to previously reported protocols (27,36). Briefly, 100 fmol of the DNA substrates were incubated in the presence of binding buffer [25 mM HEPES (pH 7.8), 5 mM MgCl_2_, 80 mM KCl, 1 mM EDTA, 1 mM DTT, 10% glycerol, 1 mg/ml bovine serum albumin (BSA)] with 100 nM purified MSH2-MSH6 complex at room temperature for 30 min along with poly dI-dC competitor DNA (20-fold excess). The detailed protocol for the purification of the complex is described (36). Reactions were resolved on a 4% polyacrylamide gel at 4°C, dried and exposed with X-ray film for autoradiography.

### Statistical analysis

Statistical analysis was performed by student’s *t*-test using R.2.7.0 software.

## RESULTS

### Cisplatin resistance following BER and MMR downregulation

**Figure 1.**
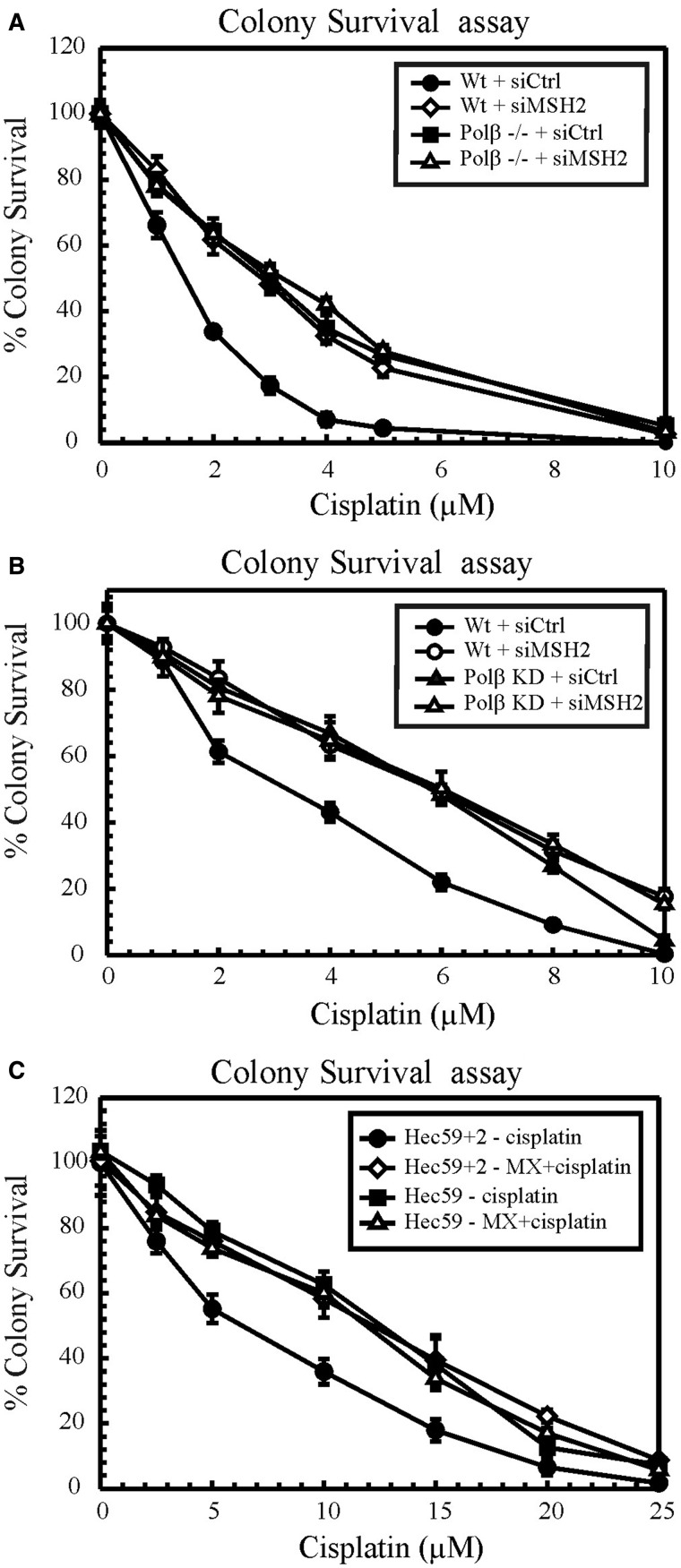
Cisplatin cytotoxicity. Colony survival assays in wild-type and Polβ null/deficient MEFs (**A**) and MDA-MB-231 cells (**B**). Control and MSH2 siRNA transfected cells were treated with increasing doses of cisplatin, and cytotoxicity was determined by clonogenic assays. (**C**) Hec59 and Hec59+2 cells were treated with cisplatin and MX+cisplatin, and clonogenic assays were performed as described. Results are represented as mean ± SD from three independent experiments.

To address the effect of BER and MMR downregulation on cell viability in response to cisplatin treatment, clonogenic assays were performed in proficient and deficient cells (Figure 1). Wild-type and Polβ null/deficient MEFs (Figure 1A) and MDA-MB-231 cells (Figure 1B) were transfected with siRNA directed against MSH2 to examine cisplatin sensitivity. Both Polβ null and Polβ deficient cells displayed cisplatin resistance compared with wild-type cells, consistent with our previous report (30). Knockdown of MSH2 in wild-type cells enhanced cisplatin resistance by ∼2-fold, which is consistent with the literature (14–17,37). However, knockdown of MSH2 in Polβ null/deficient cells did not give rise to any additional resistance to cisplatin (Figure 1A and B). To confirm these results, we used MX to inhibit BER in MSH2-proficient and -deficient cells (Figure 1C). MX binds to abasic sites and hinders Ape1 cleavage and therefore leads to the inhibition of BER (38). In parallel with previous reports, MSH2-deficient Hec59 cells showed cisplatin-resistant phenotypes compared with their proficient counterparts (14–17). In MSH2-proficient Hec59+2 (Figure 1C) cells, MX treatment led to cisplatin resistance. We recently showed that MX treatment results in a cisplatin-resistant phenotype in MEFs and human cancer cells (30). However, MMR-deficient cells, when treated with MX, exhibited no increased resistance to cisplatin. If BER and MMR operate in separate mechanistic pathways, an additive response in cisplatin resistance would be expected. The failure to observe an additive effect provides evidence that BER and MMR have epistatic roles in mediating cisplatin cytotoxicity.

### Cisplatin intrastrand adduct repair following BER and MMR downregulation

Cisplatin cytotoxicity is mediated by the formation of DNA intrastrand adducts and ICLs. Resistance develops when there is an increased repair of these adducts, as the longer the adducts persist in the DNA, the higher the sensitivity to cisplatin (39). Therefore, we assessed the repair kinetics of cisplatin DNA adducts to understand the mechanism of chemoresistance we observed from the clonogenic experiments. Studies show that MMR proteins bind to cisplatin-GG intrastrand adducts (20,21,40,41), but are not involved in the repair of the adducts. Defects in the MMR pathway contribute to increased replicative bypass of these platinum adducts (42). Recently, we showed that BER is not involved in the repair of cisplatin-GG intrastrand adducts (30), although *in vitro* studies indicate that Polβ can catalyze translesion synthesis past cisplatin-GG adducts (7,31). Therefore, we evaluated the repair kinetics of cisplatin-GG intrastrand adducts over a period following BER and MMR downregulation. We used an enzyme-linked immunosorbent assay method using a monoclonal antibody specific for cisplatin-GG adducts and calculated the percentage of adducts remaining over time, relative to the percentage of adducts present at 0 h (100%; 2 h post cisplatin treatment). Loss of BER and/or MMR showed no significant difference in the rate of intrastrand adduct removal compared with the proficient cells (Supplementary Figure S3). Cenni *et al.* (43) have shown that the repair kinetics of total platinum adduct removal was similar in MMR-proficient and -deficient cells. These data indicate that both BER and MMR pathways do not contribute to the measurable repair of cisplatin-GG intrastrand adducts.

### Cisplatin ICL repair following BER and MMR downregulation

**Figure 2.**
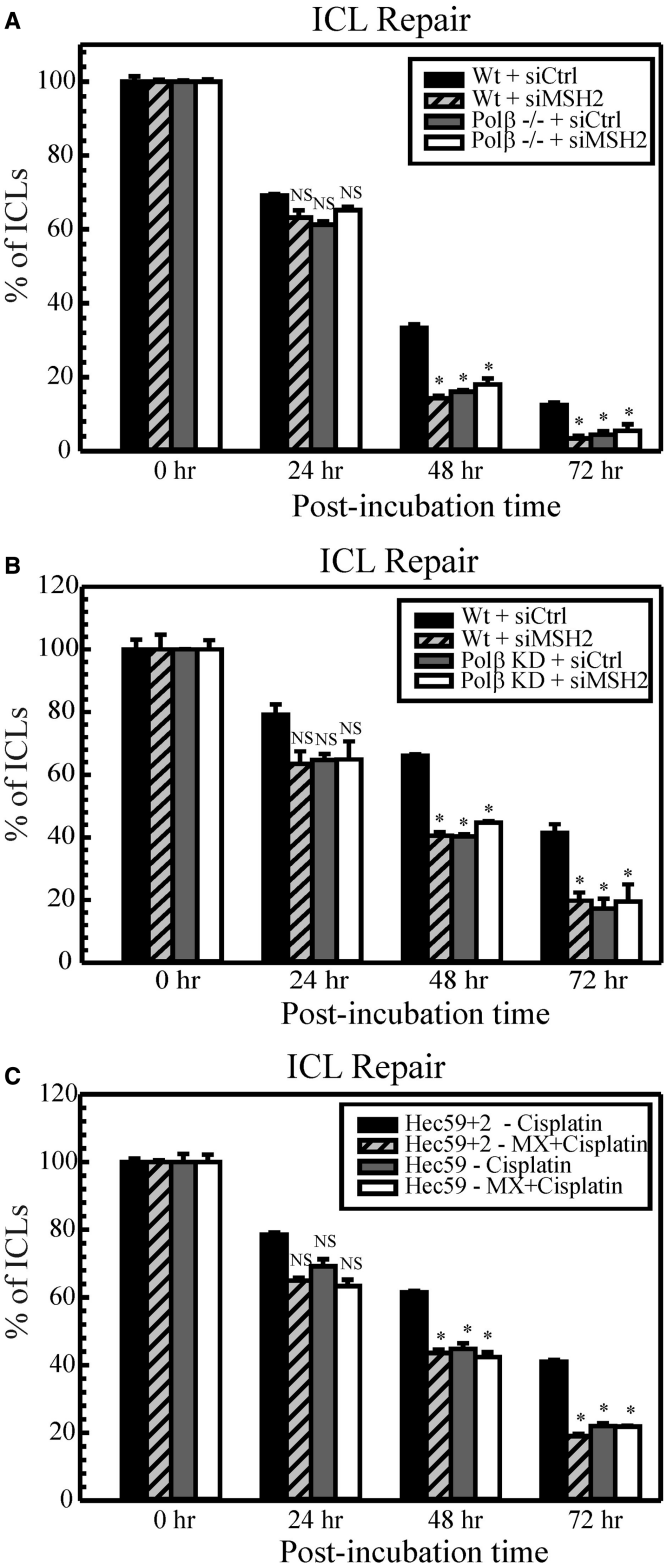
Repair of cisplatin ICLs in MEFs (**A**), MDA-MB-231 (**B**) and human endometrial (**C**) cells. Cells were treated with cisplatin, and comet assays were performed as described at different time intervals (0, 24, 48 and 72 h). The percentage of ICLs present at each time point was calculated using olive tail moments. Results are represented as mean ± SD of three independent experiments. Statistical analysis was performed by student’s *t*-test, and comparisons are made between wild-type and proficient cells versus deficient cells. NS, non-significant; *P < 0.05.

As cisplatin intrastrand adduct repair is unaffected during defective BER and/or MMR, we addressed whether cisplatin ICL repair could contribute to the observed cisplatin resistance. We used an alkaline comet assay to evaluate the repair kinetics of cisplatin ICLs over a period following BER and MMR downregulation (Figure 2). In wild-type proficient cells, cisplatin ICLs were repaired efficiently from 0 to 72 h. At 24 h, we observed no significant difference in ICL removal when comparing the proficient and deficient cells. However, at the 48 and 72 h time point, Polβ null MEFs (Figure 2A) and Polβ deficient MDA-MB-231 (Figure 2B) cells as well as MX-treated Hec59+2 (Figure 2C) cells showed a significant decrease in the percentage of ICLs, consistent with our previous report (30). Depletion of MSH2 in human cancer cells as well as MEFs resulted in enhanced repair of cisplatin ICLs at 48 and 72 h. This suggests that a faster repair of cisplatin ICLs in MMR-deficient cells contributes to enhanced cisplatin resistance. Recent studies demonstrating binding of MMR proteins to cisplatin ICLs indicate a possible role for these repair proteins in ICL processing (26,27). Cells defective in both BER and MMR displayed a similar trend in ICL repair compared with cells defective in BER or MMR alone. There was no additional ICL repair capacity when both repair pathways are targeted together. These data are also consistent with results from γH2AX foci (Supplementary Figure S4). The histone variant H2AX is phosphorylated at ser139 (γH2AX) in response to DNA-damaging agents and forms distinct nuclear foci at sites of double strand breaks (DSBs) (44). Cisplatin ICL processing leads to the generation of DSBs and therefore forms γH2AX foci (45). Loss of γH2AX foci thus correlates with the repair of ICLs, and increased DSB repair indicates an elevated resistance to cisplatin (30,44). The rate of DSB repair parallels the rate of ICL repair as seen in Figure 2 (Supplementary Figure S4). This implies that BER and MMR function within the same mechanistic pathway to mediate cisplatin sensitivity via blocking the repair of cisplatin ICLs.

### Polβ polymerase activity mediates cisplatin cytotoxicity

**Figure 3.**
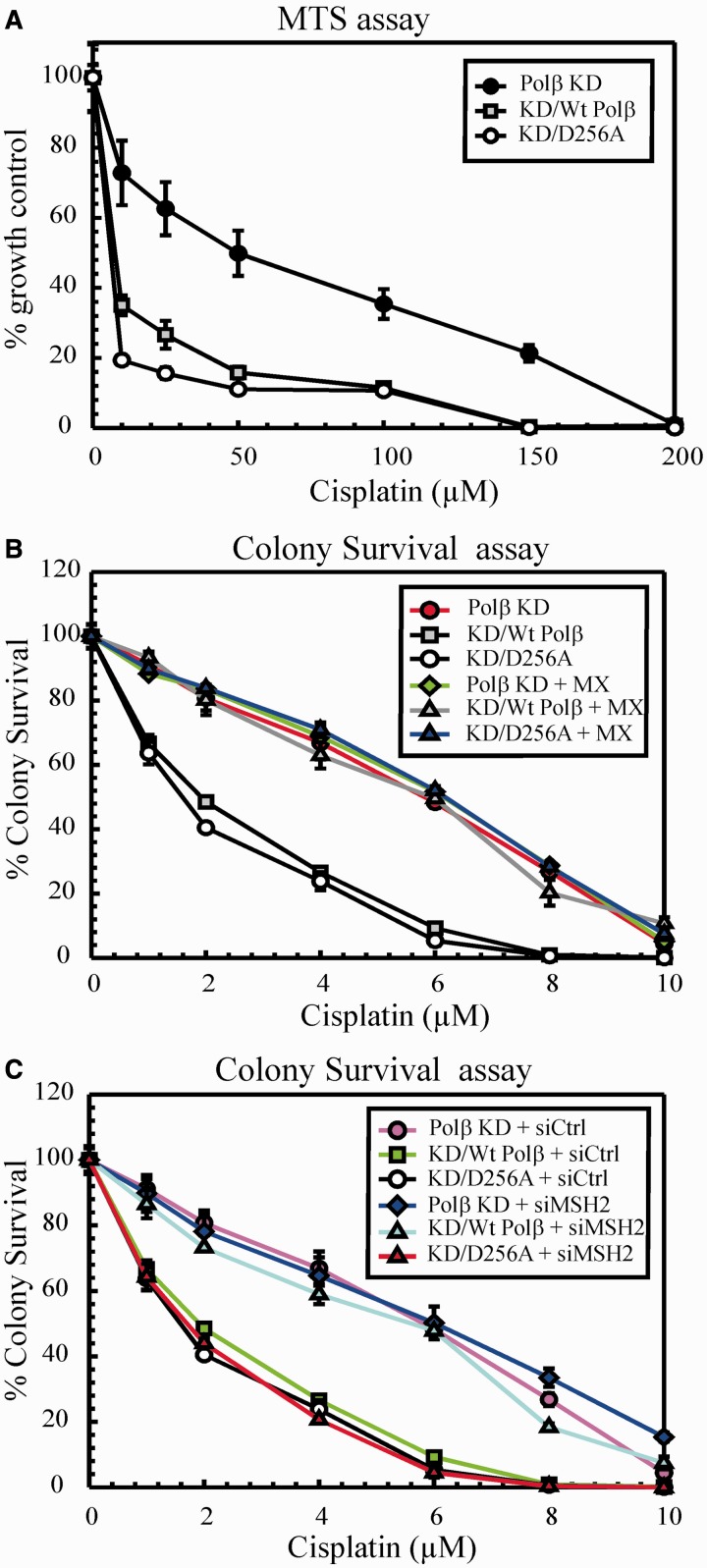
Cisplatin cytotoxicity. (**A**) Polβ KD, KD/wt Polβ and KD/D256A cells were treated with cisplatin for 2 h, and then cell sensitivity was determined by MTS assay. (**B**) Cells were treated with cisplatin and MX+cisplatin, and clonogenic assays were performed as described. (**C**) Cells were transfected with control or MSH2 siRNA, and cisplatin cytotoxicity was determined by clonogenic assays. Results are represented as mean ± SD from three independent experiments.

DNA Polβ is a 39 kDa single polypeptide consisting of a 8 kDa dRP lyase domain and a 31 kDa polymerase domain (46,47). Asp256 is one of the three active site aspartates in the polymerase domain, which is critical for the nucleotidyltransferase mechanism. Menge *et al.* (48) showed that mutation of Asp256 to alanine (D256A) completely abolished the polymerase activity of Polβ. Although the D256A Polβ mutant completely lacks the gap-filling DNA synthesis, it retains the dRP lyase activity (49). Several mutations in polymerase or the lyase domain of Polβ showed the importance of these domains in the cytotoxicity of certain DNA-damaging agents (49–55). Therefore, we re-expressed wild-type Polβ and a variant deficient in polymerase activity (D256A) in Polβ knockdown cells to determine the importance of the polymerase domain/activity in mediating cisplatin sensitivity. Consistent with a previous report, Polβ KD cells displayed cisplatin resistance (30). Re-expressing wild-type Polβ in the KD cells restored cisplatin sensitivity in both MTS (Figure 3A) and colony survival assays (Figure 3B). Interestingly, expression of the D256A Polβ mutant in the KD cells resulted in sensitivity to cisplatin as well. These data indicate that loss of Polβ protein can drive cisplatin resistance, but re-expression of wildtype or the presence of a polymerase dead mutant can mediate cisplatin sensitivity.

To assess the influence of targeting BER upstream of Polβ, we treated the cells with MX, an inhibitor of Ape1 (Figure 3B) (38). In our previous report, we showed that MX confers resistance to cisplatin treatment in a variety of cells (30). Polβ KD cells treated with MX did not show any further cisplatin resistance, indicating that MX targets the same mechanism that mediates cisplatin sensitivity. However, re-expressed wild-type Polβ and D256A cells showed a cisplatin resistant phenotype with MX treatment. These data show that targeting BER upstream of Polβ in both re-expressed wild-type and D256A cells can still mediate cisplatin cytotoxicity.

Considering that loss of Polβ protein results in a cisplatin-resistant phenotype, we wanted to dissect the mechanism of cisplatin sensitivity in the polymerase dead D256A mutant cells. Studies suggest that Polβ variants accumulate BER intermediates such as single nucleotide gaps and strand breaks owing to incomplete BER (50,53,54). Therefore, we measured single-strand breaks (SSBs) on cisplatin treatment by alkaline comet assay (Supplementary Figure S5A). Treatment of cisplatin for 2 h did not show any difference in the SSB formation (data not shown). Therefore, we treated the cells with cisplatin for 24 h and found that D256A generated ∼4–5-fold more SSBs compared with wild-type and KD cells. MX which binds to abasic sites and prevents Ape1 incision completely abolished the induction of SSBs in all three cell lines tested. To corroborate the results, we performed MTS assays with 24 h cisplatin treatment (Supplementary Figure S5B). We observed that D256A cells were hypersensitive to cisplatin compared with wild-type cells. Annexin V staining (Supplementary Figure S5C) also correlated with these results showing enhanced apoptosis in D256A cells. Collectively, these results indicate that cisplatin treatment results in accumulation of SSBs, which leads to cell death and enhanced cisplatin cell sensitivity in Polβ D256A mutant cells.

### MMR requires Polβ polymerase activity to mediate cisplatin cytotoxicity

After establishing the requirement of Polβ protein in mediating cisplatin cytotoxicity, we wanted to investigate the involvement of MMR in the cisplatin response in the context of wild-type and D256A mutant Polβ. Our previous work demonstrated that Polβ is mutagenic at sites flanking a cisplatin ICL (30). Thus, we hypothesized that MMR involvement in mediating cisplatin cytotoxicity would be dependent on Polβ misincorporation and ultimately active polymerase activity (model, Figure 6). Therefore, we transfected Polβ KD, KD/wt and KD/D256A cells with MSH2 siRNA and monitored the cell survival by clonogenic assays (Figure 3C). Consistent with our previous study (30) and as seen in Figure 1A and B, KD/wt Polβ cells showed cisplatin resistance following MSH2 knockdown. Polβ KD cells following MSH2 knockdown did not show any additional cisplatin resistance, which is consistent with an epistatic role of BER and MMR in mediating cisplatin cytotoxicity. Interestingly, knockdown of MSH2 in Polβ D256A mutant cells did not elicit cisplatin resistance. These data clearly demonstrate that MMR acts downstream of BER in mediating cisplatin cytotoxicity, and the involvement of MMR in this mechanism is dependent on Polβ polymerase activity and likely dependent on misincorporation at cisplatin ICL processed sites.

**Figure 4.**
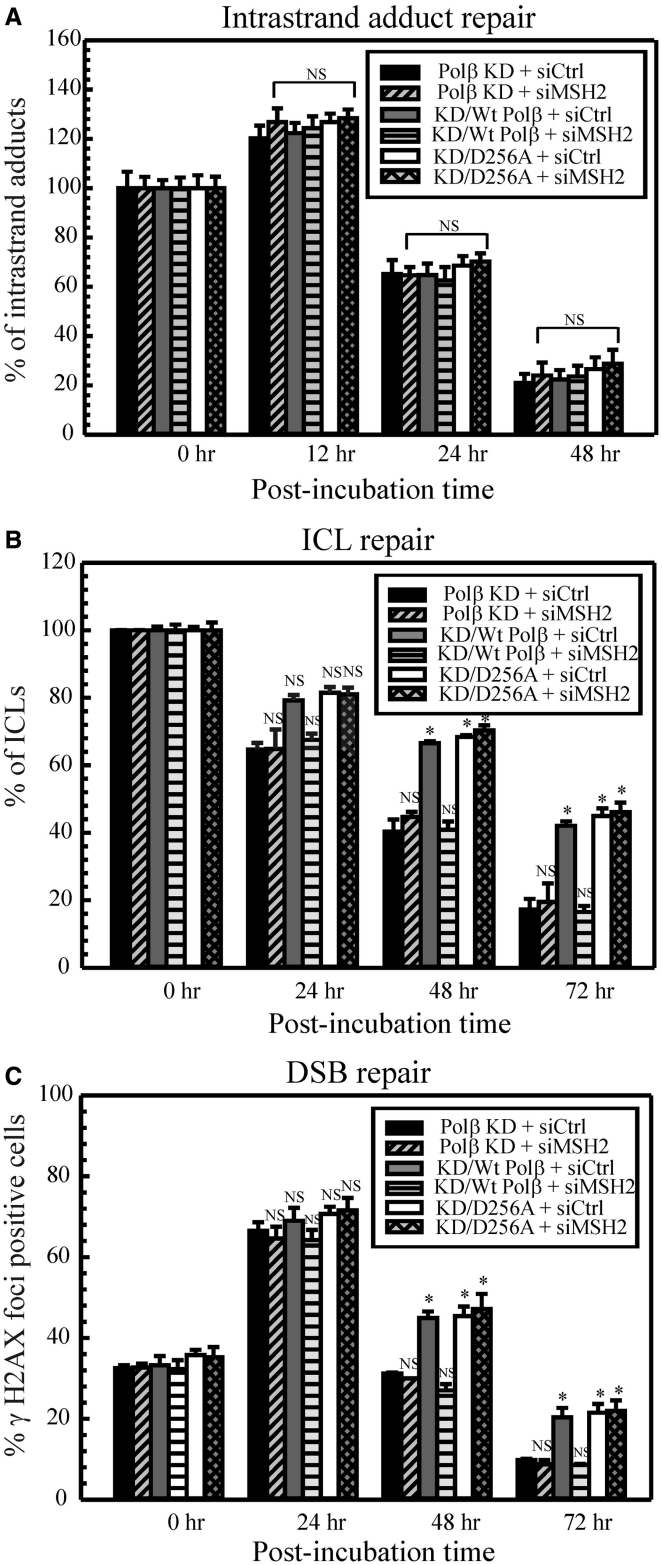
Repair of cisplatin–DNA lesions. Polβ KD, KD/wt Polβ and KD/D256A cells were transfected with control or MSH2 siRNA and the repair kinetics of cisplatin intrastrand adducts (**A**), cisplatin ICLs (**B**) and cisplatin ICL-induced DSBs (**C**) were assessed as described in ‘Materials and Methods’ section. Results are represented as mean ± SD of three independent experiments. Statistical analysis was performed by student’s *t*-test, and comparisons are made between wild-type and proficient cells versus deficient cells. NS, non-significant; *P < 0.01.

DNA repair assays in D256A mutant cells reflect what was observed in the cell survival assays (Figure 4). Cisplatin intrastrand adduct repair was unaffected in D256A cells and similar to Supplementary Figure S3 in all cell lines as expected (Figure 4A). Polβ KD cells displayed faster cisplatin ICL repair (Figure 4B) and DSB repair (Figure 4C), whereas KD/wt Polβ cells exhibited a restored inhibition of cisplatin ICL and DSB repair. Cisplatin ICL repair in the D256A mutant cells was slower than for the Polβ KD cells and similar to control wild-type cells. Consistent with the cell survival assays, there was no difference in the ICL or DSB repair in KD/D256A cells with or without MSH2 knockdown (Figure 4B and C). These data further strengthen the finding that MMR activation in mediating cisplatin cytotoxicity is dependent on the polymerase activity of Polβ.

### *In vitro* interaction of MMR proteins with cisplatin ICL DNA containing a mismatch

**Figure 5.**
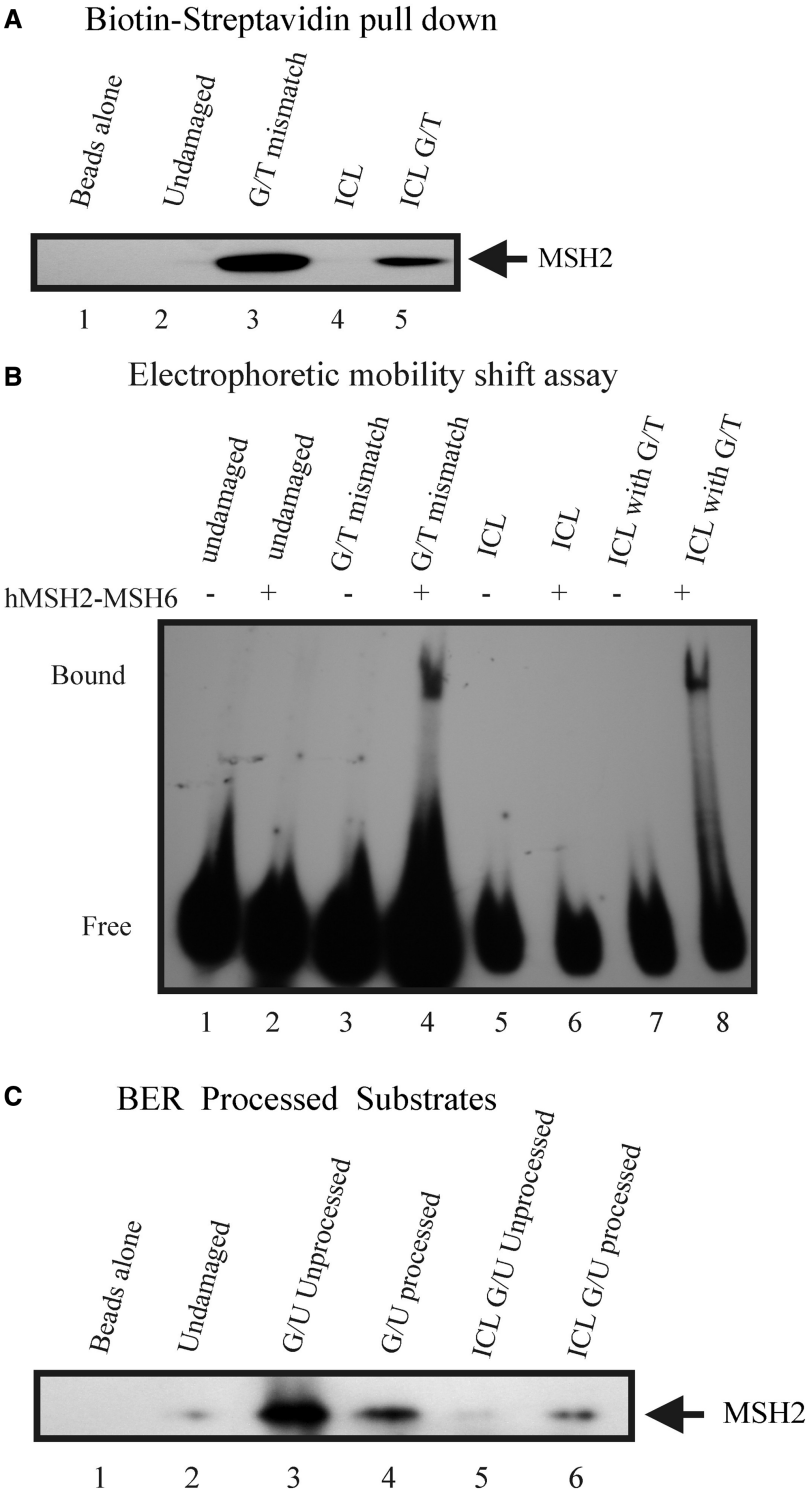
Interaction of MSH2 with cisplatin ICL DNA containing a mismatch. (**A**) Undamaged and single cisplatin ICL containing duplexes with and without a mismatch were bound to streptavidin magnetic beads and were incubated with equal amounts of overexpressed hMSH2-hMSH6 insect cell extract. The proteins bound to the DNA beads were eluted and immunoblotted with the antibody against MSH2. (**B**) EMSAs of hMSH2-hMSH6 binding to undamaged duplex DNA, G/T mismatch, cisplatin ICL and cisplatin ICL G/T substrates. (**C**) Duplex biotinylated 42 mers containing a uracil in undamaged DNA substrates or adjacent to a cisplatin ICL were processed with UDG, Ape1 and Polβ before carrying out similar pull-down assays.

To confirm the aforementioned hypothesis, we assessed the possibility that MMR binding occurs after Polβ misincorporation at sites flanking a cisplatin ICL (Figure 5). To test this, we prepared 42-mer biotinylated synthetic oligonucleotides containing a single cisplatin ICL and also a cisplatin ICL containing a mismatch adjacent to the cross-link, which would mimic Polβ misincorporation. We used substrates containing a mismatch (G/T) and undamaged duplexes as controls. We prepared these substrates as described in our previous reports (30,35). We conducted biotinylated-DNA-streptavidin pull down experiments as described in the ‘Materials and Methods’ section. This experiment allows us to monitor the relative retention of MSH2 on each DNA substrate following incubation of the substrates with insect cell extract over-expressing hMSH2-hMSH6. In Figure 5A, control experiments demonstrate that MSH2 binds a G/T mismatched DNA substrate (lane 3), whereas no retention is observed on the undamaged DNA substrate under these conditions (lane 2). MSH2 is preferentially retained on the DNA substrate containing a cisplatin ICL with a mismatch (lane 5) compared with no observed binding of MSH2 on the cisplatin ICL substrate (lane 4). These data suggest that MMR binding and subsequent activation can occur only when a mismatch is created during the processing of DNA flanking cisplatin ICLs. We also tested the ability of purified hMSH2-MSH6 to bind to DNA substrates using EMSAs (Figure 5B). Similar to the pull-down experiments, hMSH2-MSH6 binds strongly to the cisplatin ICL substrate containing a mismatch (lane 8). Little binding of hMSH2-MSH6 is observed on the cisplatin ICL substrate under these conditions (lane 6) confirming the results of the pull-down experiment. Taken together, our results suggest that MMR binding occurs downstream of a processing event that generates DNA mismatches during cisplatin ICL DNA repair. To test the possibility that binding of MMR could occur as a result of nucleotide misincorporation by Polβ, we generated undamaged uracil and cisplatin ICL DNA substrates containing a uracil adjacent to the cross-link. The substrates were processed with UDG, Ape1 and DNA Polβ in the presence of dNTPs. This reaction would result in the removal of uracil, followed by cleavage at the abasic site and subsequent Polβ incorporation of nucleotides at the uracil site. Our previous studies demonstrated that DNA Polβ could incorporate incorrect nucleotides even in the presence of correct nucleotides (30). We used these processed DNA substrates and carried out biotinylated-DNA-streptavidin pull-down experiments. As observed in Figure 5C, higher retention of MSH2 was observed on the undamaged uracil containing substrate compared with the undamaged processed DNA substrate (compare lane 3 with lane 4). This is consistent with previous studies demonstrating that hMSH2-MSH6 can bind a G-U mismatch with high affinity (56). Following BER processing, Polβ can incorporate the correct nucleotide in the undamaged DNA substrate, which results in less hMSH2-MSH6 binding. In the cisplatin ICL containing DNA, however, hMSH2-MSH6 binding is significantly enhanced following BER processing (compare lane 6 with lane 5), suggesting that enhanced MMR protein binding occurs as a result of Polβ misincorporation at sites adjacent to the cisplatin ICL.

**Figure 6.**
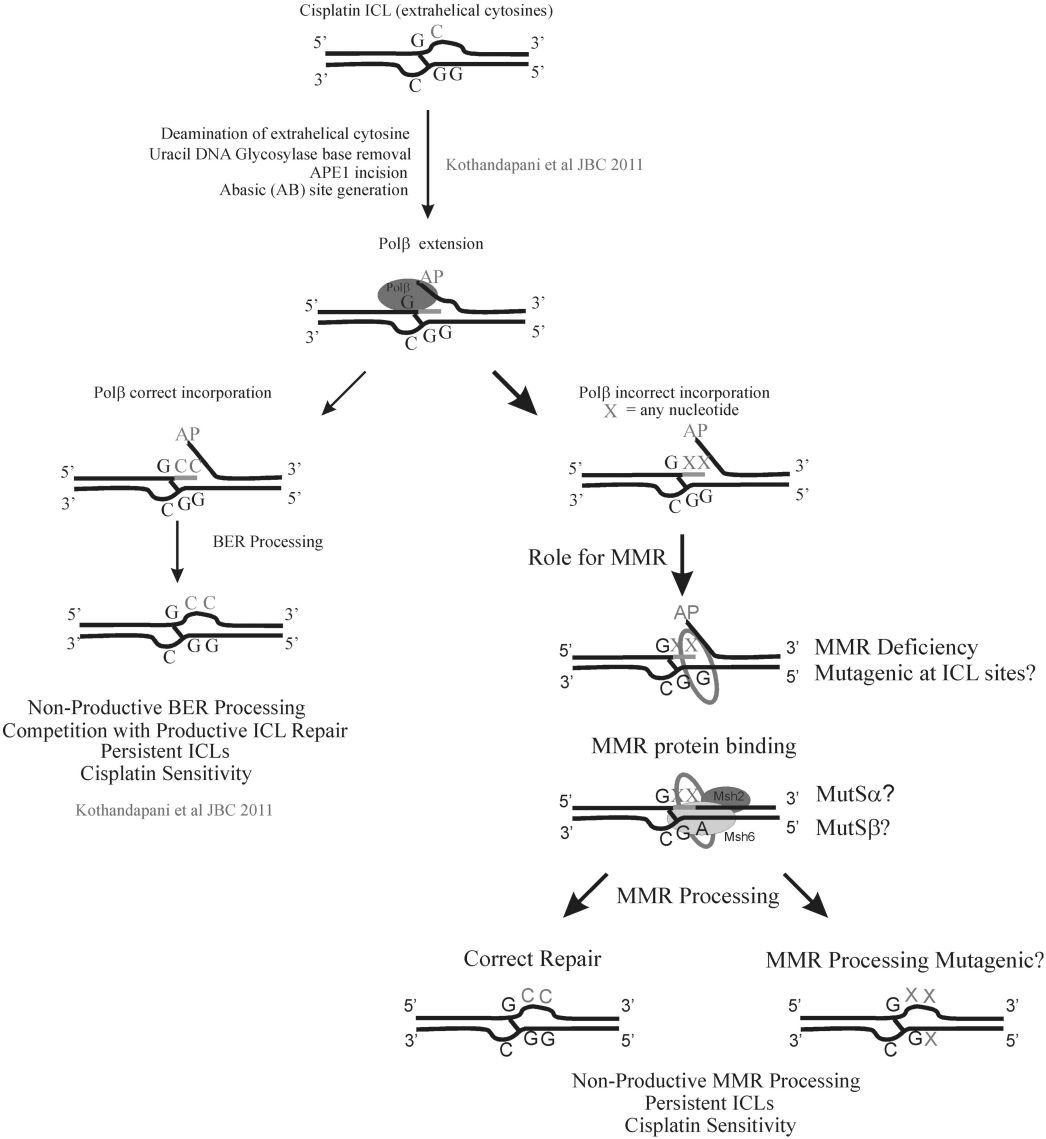
BER and MMR play an epistatic role in mediating cisplatin cytotoxicity. BER processes the DNA flanking the ICL and Polβ produces a mutagenic spectrum at the incision site (30). Incorporation of incorrect nucleotides would generate mismatched bases and act as a nucleation point for MMR protein binding and further processing. Both BER and MMR processing would result in non-productive repair of cisplatin ICLs. As a result of blocking productive ICL repair pathways, there are persistent cisplatin ICLs, which enhance cisplatin cytotoxicity.

Our results show that MMR functions downstream of BER cisplatin ICL processing, and MMR activation in mediating cisplatin cytotoxicity is dependent on the polymerase activity of Polβ (Figure 6). This is the first report that demonstrates that MMR and BER play an epistatic role in mediating cisplatin cytotoxicity and a direct connection between MMR involvement and Polβ polymerase activity.

## DISCUSSION

Cisplatin is one of the most widely used anti-cancer agents. Despite the favorable initial response of tumors to cisplatin chemotherapy, remission and eventually resistance becomes a major impediment. Several lines of evidence indicate that cisplatin resistance can be attributed to increased DNA repair (39). Interestingly, deficiency or loss of MMR components results in cisplatin resistance. Although several mechanisms have been postulated, the exact mechanism underlying the resistant phenotype is not clear (14–17). We have recently shown that defective BER also contributes to cisplatin resistance (30). The mutational spectrum exhibited by Polβ across the cisplatin DNA adducts indicates a possible involvement of MMR in processing the DNA that flanks these cisplatin adducts (30,31,42,57). Therefore, we studied the effect of downregulating BER and MMR on cisplatin cytotoxicity in human cancer cells and MEFs to address the potential connection of these two DNA repair pathways in mediating a response to cisplatin treatment.

Clonogenic assays in our study revealed that depletion of BER or MMR resulted in cisplatin resistance as expected. Interestingly, when both BER and MMR pathways are targeted, there was no additional resistance to cisplatin. Liu *et al.* (58) showed that disrupting BER by MX treatment enhanced sensitivity to methylating and DNA cross-linking agents in MMR-proficient as well as MMR-deficient cells (59). Recent synthetic lethality studies show that disrupting BER by downregulation of DNA polymerases can be a potential target in MMR-deficient cancers (60). In the current study, MX treatment as well as Polβ deficiency displayed neither hypersensitivity nor enhanced resistance to cisplatin treatment in MSH2-deficient cells. The ∼2-fold cisplatin resistance observed following BER or MMR downregulation remained the same following downregulation of both DNA repair pathways. Therefore, we hypothesize that BER and MMR pathways function together in the same mechanistic pathway to mediate cisplatin cytotoxicity. This is the first report showing a possible epistatic role of BER and MMR in mediating cisplatin sensitivity in mammalian cells.

Studies suggest that the MMR system functions as a cisplatin–DNA damage sensor, as MutS and hMutSα preferentially recognize cisplatin intrastrand adducts (26,41,61,62). Assessment of cisplatin 1,2 dGpG intrastrand adduct repair revealed that BER as well as MMR are not involved in the removal of these adducts, which indicates that the increased resistance is not due to increased repair of cisplatin intrastrand adducts (Supplementary Figure S3). MSH2 has been shown to be involved in the recognition and processing of psoralen ICL DNA substrates, and MSH2 deficient cells are hypersensitive to psoralen and display reduced ICL-induced repair synthesis (63). In our study, deficiency of MSH2 resulted in increased repair of cisplatin–ICLs and ICL-induced DSBs. This implies that ICL processing events vary with different cross-linking agents and that enhanced ICL repair contributes to MMR-mediated cisplatin resistance. As stated earlier, several mechanisms have been proposed for MMR-mediated cisplatin resistance. In this report, we show that cisplatin ICL repair contributes to resistance in human cancer cells as well as MEFs following loss of MMR. Of importance, an additive response was not observed in the ICL and DSB repair when targeting both BER and MMR pathways. The fold increase in the repair of these lesions remained the same during BER and/or MMR downregulation, demonstrating that BER and MMR mediate cisplatin cytotoxicity by affecting cisplatin ICL DNA repair.

Mutations in Polβ and the resulting variants have been reported in several tumors (52,55,64,65). The Polβ 249-262 deletion variant is reported in colorectal cancers (66), and therefore, the mutant variant used in our study (D256A) has clinical significance. Using Polβ null cells re-expressing the D256A variant, Sobol *et al.* (49) have shown that DNA synthesis activity of Polβ is not essential for MMS cytotoxicity. Here, we show that Polβ protein is essential for mediating cisplatin cytotoxicity, and the polymerase activity is required for the activation of MMR in mediating cisplatin response. Polymerase activity has also been shown to be critical for survival (53). In BER, Polβ catalyzes DNA synthesis by filling the single nucleotide gap following Ape1 incision. The D256A mutant lacks polymerase activity and therefore results in incomplete BER after cisplatin treatment. The unfilled gaps accumulate into SSBs, which ultimately lead to cell death. The reduced induction of SSBs in Polβ KD cells could be due to repair of these strand breaks by other DNA polymerases or other DNA repair pathways such as NER, homologous recombination and single strand break repair (SSBR). Lang *et al.* (50) have shown that a polymerase mutant E295K binds to the single nucleotide gap and precludes wild-type Polβ from accessing the gap. Consistent with this, D256A could be a dominant negative mutant, which competes with wild-type Polβ and the inability to fill the gap could eventually result in cell death owing to accumulation of SSBs. In this scenario, the D256A mutant could still block productive cisplatin ICL DNA repair and lead to cell sensitivity, which is consistent with our results. In support of this, BER processing upstream of Polβ is intact in the D256A mutant cells, and if this processing is blocked by MX, cisplatin resistance is still observed.

Our previous results demonstrated that Polβ is mutagenic at sites flanking a cisplatin ICL; thus, we assessed the requirement of Polβ polymerase activity in MMR activation in response to cisplatin (30). We downregulated MSH2 in Polβ KD, KD/wt Polβ and KD/D256A cells. As expected, wild-type cells displayed cisplatin sensitivity, and Polβ KD cells as well as MSH2 knockdown resulted in cisplatin resistance. However, knockdown of MSH2 in the Polβ D256A mutant cells showed no additional effect on cell sensitivity or cisplatin DNA repair. This is an important observation, as these data provide compelling evidence for a direct role of Polβ polymerase activity in activating MMR to mediate cisplatin cytotoxicity. Consistent with these data, we observed that hMSH2-MSH6 binds weakly to a cisplatin ICL DNA unless a mismatch is present adjacent to the cisplatin ICL or the DNA substrate is processed by the BER machinery.

Our previous report (30) demonstrated a novel role of BER in processing a uracil adjacent to the cisplatin ICL, which is a likely cellular product owing to preferential deamination of the extrahelical cytosines induced by the cisplatin ICL. Uracil is an efficient substrate for the BER machinery and if the processing by BER and subsequent nucleotide incorporation by Polβ culminates in the generation of a mismatch, this site would serve as an entry point for the MMR proteins to influence cisplatin cytotoxicity by competing with and inhibiting the repair of cisplatin ICLs (Figure 6). This is contrary to previously proposed models in which MMR was suggested to play a role in cisplatin intrastrand adduct binding and subsequently result in either ‘futile cycle’ DNA repair, damage shielding or damage signaling to mediate cisplatin cytotoxicity. Our findings that MMR acts downstream of BER and is dependent on Polβ polymerase activity in mediating cisplatin cytotoxicity will result in a paradigm shift in the cisplatin DNA repair field, as these results highlight a novel mechanism of mediating cisplatin cytotoxicity via non-productive ICL processing.

In conclusion, based on our results, we propose that BER and MMR function in the same mechanistic pathway required to sustain cisplatin sensitivity. BER processing events at sites adjacent to cisplatin ICLs can result in incorporation of correct as well as incorrect bases adjacent to the ICL (30). Nucleotide misincorporation by Polβ during BER processing at sites flanking a cisplatin ICL would generate mismatched bases and likely result in MMR protein binding and further MMR processing (Figure 6). These events block productive cisplatin ICL repair pathways and result in persistent cisplatin ICLs, which lead to cell sensitivity. It will be interesting to delineate the specific roles MutSα, MutSβ and MLH1 play in cisplatin ICL processing and determine how MMR affects the mutation spectrum at the DNA flanking the cisplatin ICL sites. Early studies in *E.coli* reported that cisplatin induces base substitution mutations at the DNA adducts (67,68). Cisplatin AG and GG adducts are identified to be potential sites for mutations, with strong mutation frequency at AG sites through AT to TA transversions. Further studies showed that GXG adducts (GAG, GCG, GTG) are also mutational hotspots (69,70). Among these, preferential mutation occurred at GCG sites frequently. As ICLs are formed between two guanines on opposite strands at 5′-GpC sites, GCG site mutations could arise from the presence of cisplatin ICLs. This is consistent with our hypothesis that BER processing of ICLs results in a mutational spectrum that drives MMR activation and processing. However, a recent study suggests that there are minimal mutations at cisplatin ICL sites (71). Therefore, further studies are warranted to explore cisplatin-specific ICL-induced base substitution mutations and validate the proposed model in BER and MMR deficient cell lines.

## SUPPLEMENTARY DATA

Supplementary Data are available at NAR Online: Supplementary Figures 1–5, Supplementary Materials and Methods and Supplementary References [72].

## FUNDING

The National Institutes of Health (NIH) [GM088249 to S.M.P.] and the NIH [CA132385; GM087798; CA148629 to R.W.S.]. Support for the UPCI Lentiviral Facility was provided to R.W.S. by the Cancer Center Support Grant from the NIH [P30 CA047904]. Funding for open access charge: NIH [R01 to S.M.P.].

*Conflict of interest statement.* R.W.S. is a Scientific Consultant for Trevigen, Inc.

## Supplementary Material

Supplementary Data
