# Supplementary material for: Epistatic role of base excision repair and mismatch repair pathways in mediating cisplatin cytotoxicity
Source: Nucleic Acids Res. 2013 Jun 12;41(15):7332–43. doi: 10.1093/nar/gkt479 (PMC3753620; doi:10.1093/nar/gkt479)
Supplement: Supplementary Data [file supp_41_15_7332__index.html]

Epistatic role of base excision repair and mismatch repair pathways in mediating cisplatin cytotoxicity — Epistatic role of base excision repair and mismatch repair pathways in mediating cisplatin cytotoxicity — Supplementary Data 

# Epistatic role of base excision repair and mismatch repair pathways in mediating cisplatin cytotoxicity

## Supplementary Data

files

**Files in this Data Supplement:**

- Supplementary Data - pdf file
