# Supplementary material for: Epistatic role of base excision repair and mismatch repair pathways in mediating cisplatin cytotoxicity
Source: Nucleic Acids Res. 2013 Jun 12;41(15):7332–43. doi: 10.1093/nar/gkt479 (PMC3753620; doi:10.1093/nar/gkt479)
Supplement: Supplementary Data [file supp_gkt479_nar-00018-d-2013-File008.pdf]

SUPPLEMENTARY DATA

Inventory of Supplementary Data

1. Supplementary Data:

Supplementary Figure S1 (western blots showing levels of MSH2 protein knockdown)

Supplementary Figure S2 (PCR analysis showing levels of MSH2 transcript knockdown)

Supplementary Figure S3 (cisplatin intrastrand adduct repair in MEFs, MDA-MB-231 and Hec59 cells)

Supplementary Figure S4 (repair of γH2AX foci in MEFs, MDA-MB-231 and Hec59 cells)

Supplementary Figure S5 (SSB formation, MTS assays and Annexin V analysis in MDA-MB-231 cells)

2. Supplementary Figure Legends

3. Supplementary Materials and Methods

4. Supplementary References

1. Supplementary Data

Supplementary Figure S1

A MSH2 Knockdown in wt MEF

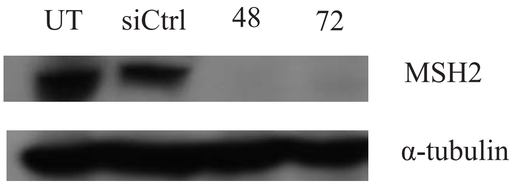

B MSH2 Knockdown in Polβ-/- MEF

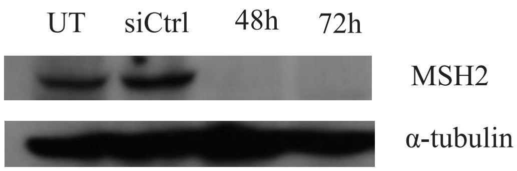

C MSH2 Knockdown in wt MDA-MB-231

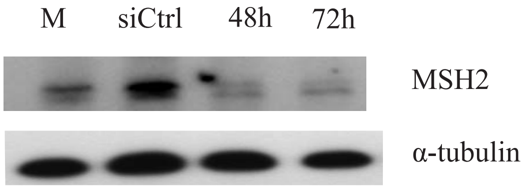

D MSH2 Knockdown in Polβ KD MDA-MB-231

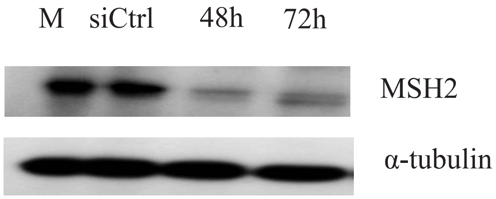

Supplementary Figure S2

A

Transcript knock-down in Polβ -/- MEFs

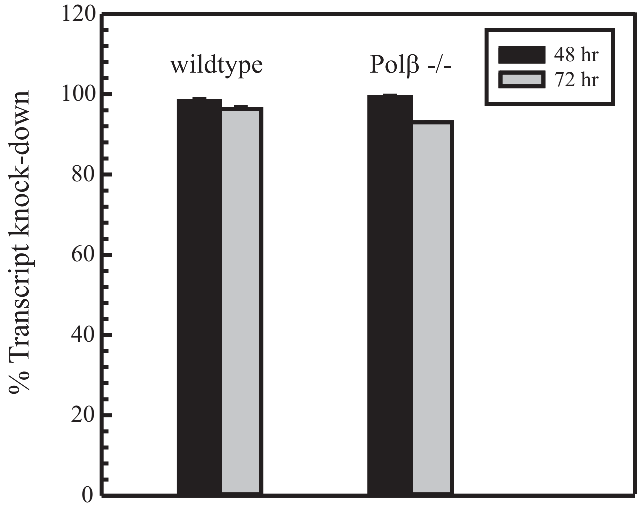

B

Transcript knock-down in Polβ KD MDA-MB-231

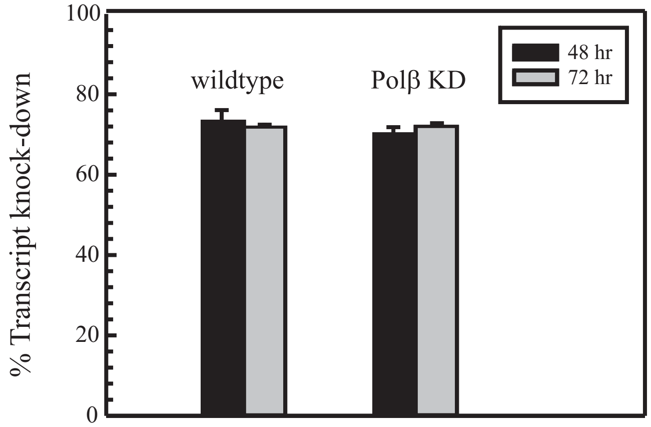

Supplementary Figure S3

A

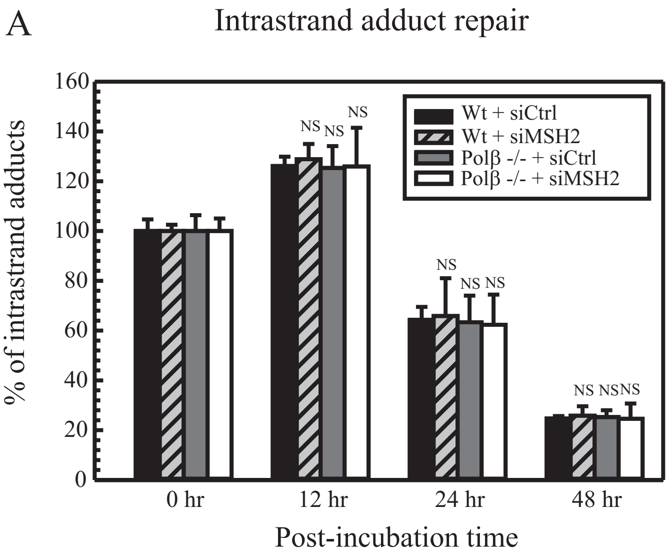

B

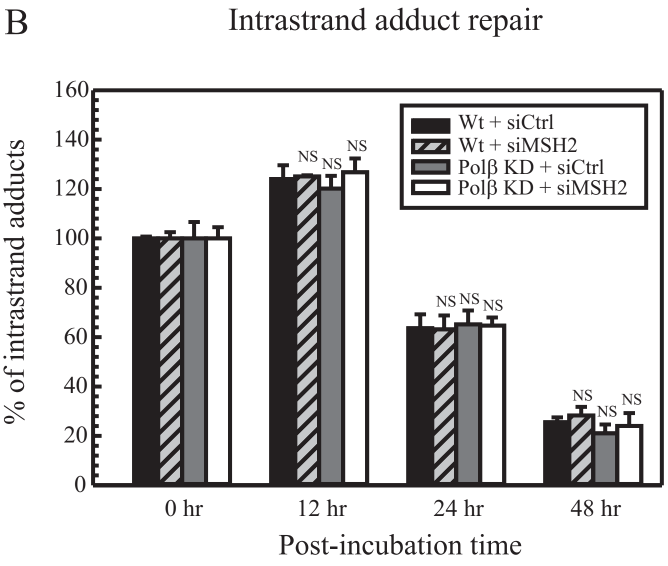

C

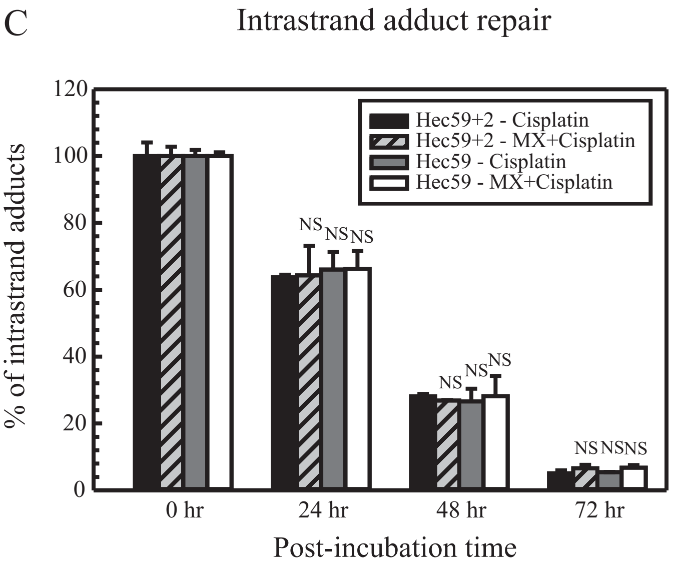

Supplementary Figure S4

A

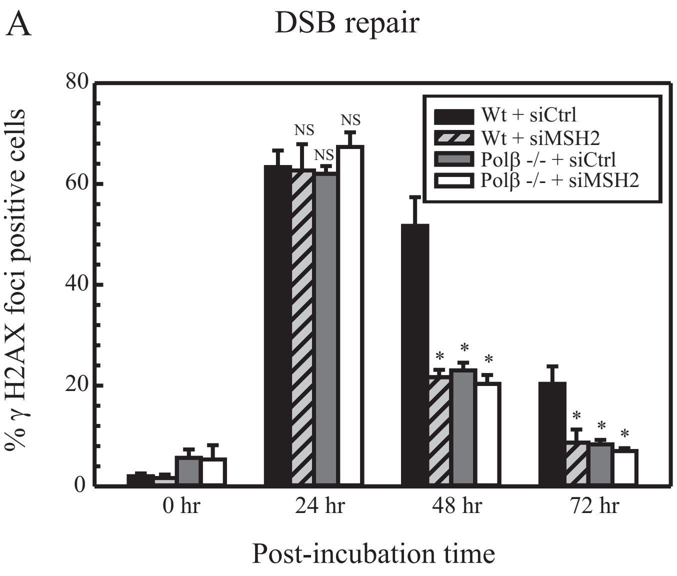

B

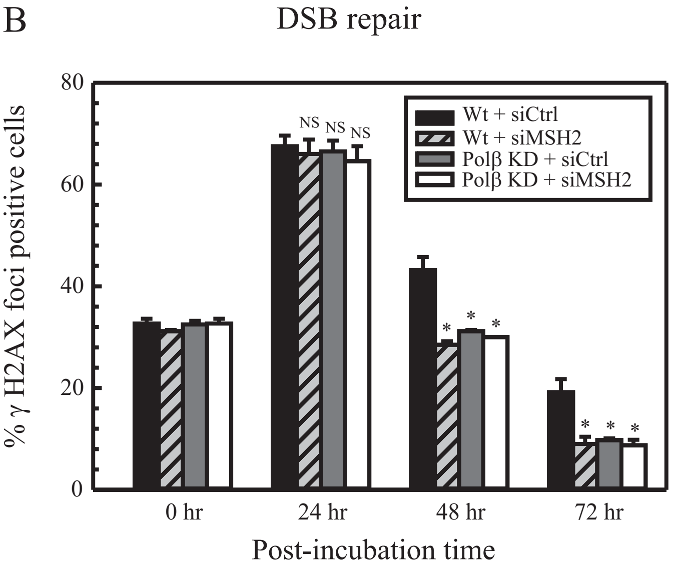

C

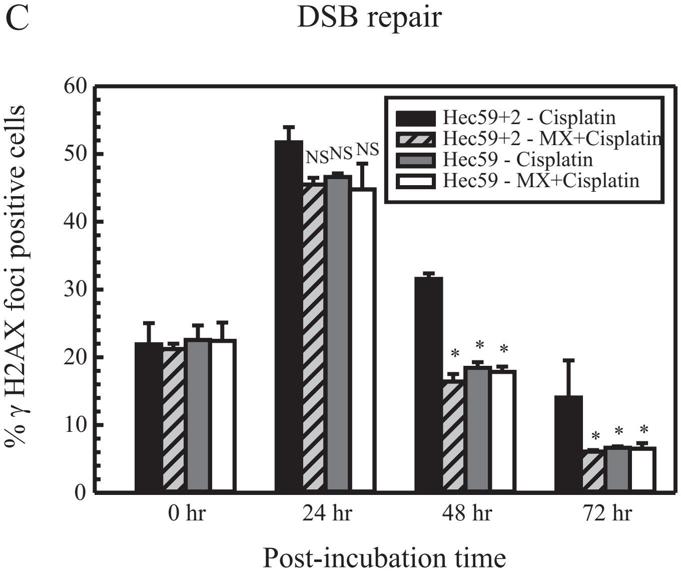

Supplementary Figure S5

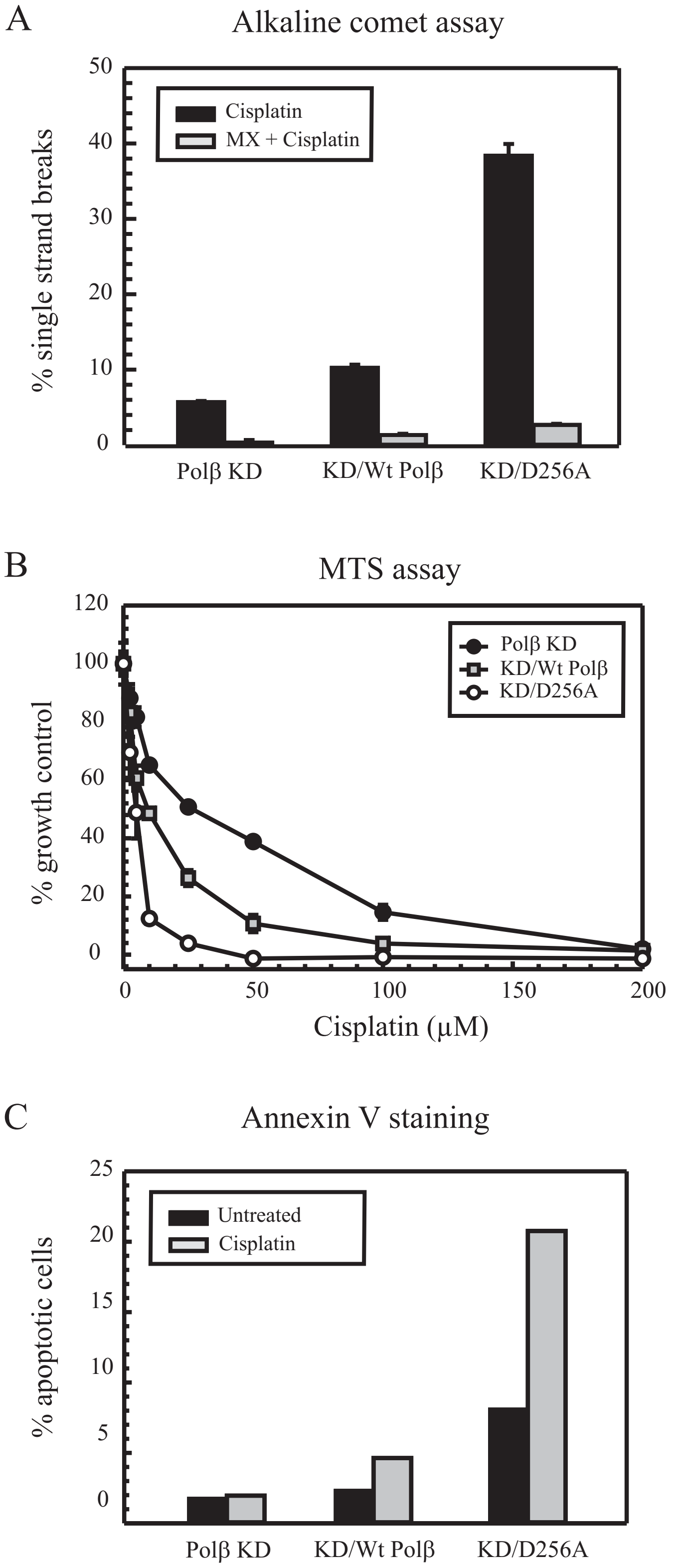

2. Supplementary Figure Legends

Supplementary Figure S1. siRNA mediated downregulation of MSH2 in wildtype (A) and Polβ null (B) MEFs, and in wildtype (C) and Polβ KD (D) MDA-MB-231 cells . Cells were transiently transfected with siRNA directed against MSH2 as described in Materials and Methods. Proteins were extracted at indicated time points and probed with MSH2 antibody with α-tubulin as loading control. Control cells were left untransfected (UT), or mock (M) and non-targeting siRNA (siCtrl) transfected.

Supplementary Figure S2. Percent transcript knockdown in wildtype and Polβ-/- MEFs (A), and in wildtype and Polβ KD MDA-MB-231 cells (B). Cells were transiently transfected with siRNA directed against MSH2. At 48 and 72 hr post transfection, total RNA was extracted from cells and the transcript levels were quantified as described in Materials and Methods with β-actin or GAPDH as an endogenous control. The percent transcript knockdown was determined from $2^{\wedge-\Delta\Delta CT}$ values with untransfected or non-targeting siRNA as controls.

Supplementary Figure S3. Repair of cisplatin intrastrand adducts in MEFs (A), MDA-MB-231 (B) and human endometrial (C) cells. Cells were treated with cisplatin and genomic DNA was isolated at the indicated time points. ELISA was performed using a cisplatin GG intrastrand adduct antibody and the percentage of GG adducts remaining was calculated as described. Results are represented as mean ± SD of three independent experiments. Statistical analysis was performed by student's t test and comparisons are made between wildtype and proficient cells vs deficient cells. NS – non-significant

Supplementary Figure S4. Repair of γH2AX foci in MEFs (A), MDA-MB-231 (B) and human endometrial (C) cells. Cells were treated with cisplatin and immunofluorescence was performed

as described at different time intervals (0, 24, 48 and 72 hr). The percentage of γH2AX foci positive cells at each time point was calculated. Results are represented as mean ± SD of three independent experiments. Statistical analysis was performed by student's t test and comparisons are made between wildtype and proficient cells vs deficient cells. NS – non-significant; * - $P < 0.05$.

Supplementary Figure S5. SSB, MTS assays and Annexin V staining in MDA-MB-231 cells. Polβ KD, KD/wt Polβ and KD/D256A cells were treated with cisplatin for 24 hr. A, % of single strand breaks was assessed by alkaline comet assay. B, cell sensitivity was determined by MTS assays. C, % of apoptotic cells were detected by the Annexin V staining method.

3. Supplementary Materials and Methods

Western blot analysis

Cells were harvested at indicated times after the first transfection, washed with PBS and lysed in lysis buffer (10 mM Tris pH 8.0, 120 mM NaCl, 0.5% NP-40, 1mM EDTA) containing protease inhibitors (0.5 M PMSF, 1mg/ml leupeptin and 1 mg/ml pepstatin A). The proteins were separated on 8% SDS-polyacrylamide gels and transferred onto Immobilon-P transfer membranes (Millipore). After blocking (2% non-fat dry milk), the membranes were probed with primary antibodies recognizing human and mouse MSH2 with α-tubulin as loading control. The membranes were incubated with appropriate secondary antibodies and the signal was detected by using Enhanced Chemiluminescence detection system.

Quantitation of transcript levels

At 48 and 72 hr post-transfection one, cells were harvested and pelleted. RNA was isolated using TRIzol reagent (Invitrogen) by standard procedures. The total RNA was reverse transcribed using MMLV reverse transcriptase enzyme (Invitrogen) as per the manufacturer's protocol. The transcript levels were quantified using iQ SYBR green supermix (Bio-Rad) in iCycler iQ System, with β-actin or GAPDH as an endogenous control. The percent transcript knockdown was determined from $2^{\wedge-\Delta\Delta CT}$ values.

Cisplatin Intrastrand adduct measurements

Cells were treated with cisplatin for 2 hr and then the repair kinetics of cisplatin intrastrand adducts was assessed by ELISA using a specific monoclonal antibody against cisplatin-intrastrand adducts (ICR4, kindly provided by Michael J. Tilby, University of Newcastle, UK) (1,2). Genomic DNA was coated on 96 well ELISA plates and probed with ICR4 antibody. After incubation with HRP conjugated goat anti-rat antibody (Calbiochem), TMB (1 step ultra TMB-ELISA, Thermo Scientific) was added. The reaction was stopped with 2M sulfuric acid and absorbance was measured at 450 nm (Spectramax M5 plate reader, Molecular Devices). The % intrastrand adducts were calculated using OD 450 nm where the 0 hr time point was used as 100% intrastrand adducts in each cell line.

γH2AX immunofluorescence

Double-strand break (DSB) repair was assessed by monitoring the nuclear γ-H2AX foci by immunofluorescence (3). Cells were fixed, permeabilized and probed with monoclonal anti γ-H2AX antibody (1:500, Millipore). The images were visualized using a Nikon Eclipse T2000-U

microscope. Foci were counted in at least 200 cells at each time point per condition in each cell line and results are expressed as % γ-H2AX foci positive nuclei.

MTS assays

The CellTiter 96 Aq_{ueous} Non-Radioactive Cell Proliferation Assay (Promega) was used to assess the cytotoxicity of cisplatin. Cells were treated with increasing doses of cisplatin for 2 hr. After 72 hr, 20 μL of combined MTS/PMS solution [(3-(4,5-dimethylthiazol-2-yl)-5-(3-carboxymethoxyphenyl)-2-(4-sulfophenyl)-2H-tetrazolium and phenazine methosulfate] was added to each well and incubated for 2-4 hr at 37°C. The absorbance was measured at 490 nm in Spectramax M5 plate reader (Molecular Devices). The percentage of cell survival was calculated from the average OD of treated cells/average OD of control cells x 100.

Single strand break measurements

Alkaline comet assay was used to measure single strand break (SSB) formation. Cell suspensions (~10000 cells) were embedded on a microscopic slide, lysed and incubated in ice-cold alkaline solution for 20 min. Electrophoresis was carried out for 25 min at 28 V, 300 mA. Slides were neutralized and stained with SYBR green (Trevigen). The comets were scored using a Nikon epifluorescence microscope. At least 100 cells were analyzed per slide and the data was expressed as the percentage of single strand breaks.

Apoptosis assays

The percentage of apoptotic cells was determined using the PE annexin V apoptosis detection kit 1 (BD biosciences Cat # 559763). In this assay, both attached and floating cells were utilized for

the analysis. Briefly, the cells were washed with cold PBS and resuspended in 1X binding buffer followed by staining with annexin V and 7AAD using FACSCalibur flow cytometer (BD Biosciences) with CellQuest software. The percentage of apoptotic cells was calculated based on the number of annexin V positive cells for all the conditions.

DNA susbtrates used

**DNA**	**Sequence (5'-3')**
42 G/T	CTCTTCCCCATCTCCTTTCCG**T**CTCCTTCCTTCCCCTTCCCT
42 ICL	CTCTTCCCCATCTCCTTTCC**GC**CTCCTTCCTTCCCCTTCCCT
42 ICL G/T	CTCTTCCCCATCTCCTTTCC**GT**CTCCTTCCTTCCCCTTCCCT
42 ICL U	CTCTTCCCCATCTCCTTT**GU**CCCTCCTTCCTTCCCCTTCCCT

The oligos were annealed to complementary DNA substrates containing a biotin modification to generate duplex DNA substrates. The position of either the mismatch or cisplatin ICL is indicated in bold.

4. Supplementary References

(1) Arora S, Kothandapani A, Tillison K, Kalman-Maltese V, Patrick SM. Downregulation of XPF-ERCC1 enhances cisplatin efficacy in cancer cells. Dna Repair 2010;9(7):745-53.

(2) Tilby MJ, Johnson C, Knox RJ, Cordell J, Roberts JJ, Dean CJ. Sensitive detection of DNA modifications induced by cisplatin and carboplatin in vitro and in vivo using a monoclonal antibody. Cancer Research 1991;51(1):123-9.

(3) Kothandapani A, Dangeti VSMN, Brown AR, Banze LA, Wang XH, Sobol RW, et al. Novel Role of Base Excision Repair in Mediating Cisplatin Cytotoxicity. Journal of Biological Chemistry 2011;286(16):14564-74.
